# 

**Published:** 1834-07-01

**Authors:** 


### CONTENTS

OF THE

### MEDICAL QUARTERLY REVIEW,

No. IV. JULY 1, 1834.

---

### REVIEWS.

I.

Pathological and Surgical Observations on the Diseases of the Joints. By B. C. BRODIE, V.P.R.S., Serjeant Surgeon to the King, and Surgeon to St. George's Hospital. Third Edition . . . 241

II.

Chemistry, Meteorology, and the Function of Digestion, considered with reference to Natural Theology. By WILLIAM PROUT, M.D., F.R.S., . 267

III.

Cours Théorique et Pratique d'Accouchemens. Par J. CAPURON, M.D. 273

IV.

The Edinburgh Medical and Surgical Journal . . 280
Dr. TURNBULL's Med.-Chirurg. Report of the Huddersfield Infirmary ib.
Mr. POOLE's Account of an Epidemic Gastric Fever . 381
Dr. CHURCHILL's Report of Diseases of Lying-in Women, &c. . ib.
Dr. HENRY on Sulphate of Magnesia . . 282
Dr. SMITH on Insanity . . . . 284
Dr. CRAIGIE's Clinical Report . . . ib.
Dr. SPITTAL on Diseases of the Heart . . 285
Dr. MALCOLM on Spontaneous Evolution of Fœtus . 286
Dr. MURRAY on the Tests for Arsenic . . 287
Mr. ROBERTON on the Relaxation, &c. of the Uterus and Bladder in the Puerperal State . . . . ib.

V.

The Anatomy and Physiology of the Liver. By FRANCIS KIERNAN, Esq. 288

VI.

On the Ulcerative Process in Joints. By C. ASTON KEY, Esq. . 256
A Practical Treatise on Diseases of the Joints. By W. J. WICKHAM, Esq. ib.

VII.

The Principles and Practice of Obstetricy, as at present taught by JAMES BLUNDELL, M.D. . . . . . 308

VIII.

Der Alp, sein Wesen und seine Heilung. Eine Monographie, von MORITZ STRAHL, Dr. der Medizin, &c. . . . 315

*Reviews continued.*

IX.

Consumption Curable; and the Manner in which Nature as well as Remedial Art operates in effecting a Healing Process in Cases of Consumption, &c. By FRANCIS HOPKINS RAMADGE, M.D. . . 320

X.

The Principles of Diagnosis. By MARSHALL HALL, M.D. . 325

XI.

Abhandlungen aus dem Gebiete der Practischen Medicin und Chirurgie. Von Dr. ADOLPH. LEOP. RICHTER, &c. . . . 331

XII.

The Anatomy of the Human Eye. By JOHN DALRYMPLE, Esq. . 340

XIII.

Leçons de Clinique Médicale, faites a l'Hôtel Dieu de Paris, par le Professeur A. F. CHOMEL; recueillies et publiées sous ses yeux, par J. L. GENEST, D.M.P. . . . . . 343

XIV.

The Transactions of the Provincial Medical and Surgical Association . 351
- Dr. BARDSLEY on the Efficacy of Strychnine in some Forms of Paralysis ib.
- Mr. THOMPSON on Chronic Peritonitis . . . 357
- Mr. DAWSON's Case of Lithotomy by the Rectum . . ib.
- Mr. GRINDROD's Case of Hydrophobia . . 360
- Mr. PRITCHARD's Tuberculous Affection of the Kidney . 361
- Mr. W. F. MORGAN on Dislocation of the Shoulder . 363
- Dr. J. K. WALKER on the Diseases of Children . . 364
- Mr. E. A. JENNINGS on the Hydrostatic Test . . 365

XV.

Principles and Illustrations of Morbid Anatomy; adapted to the Elements of M. ANDRAL, and to the Cyclopædia of Practical Medicine, &c. By J. HOPE, M.D., F.R.S. . . . . . 367

XVI.

An Inquiry into the Principles and Practice of Medicine, founded on original Physiological Investigations. By G. CALVERT HOLLAND, M.D., &c. 370

XVII.

A New Synopsis of Nosology, founded on the Principles of Pathological Anatomy, and of the Natural Affinities of Diseases. By G. HUME WEATHERHEAD, M.D. . . . . 383

XVIII.

Hortus Medicus, or Figures and Descriptions of the more important Plants used in Medicine, or possessed of Poisonous Qualities; with their Medical Properties, Chemical Analysis, &c. &c. By GEORGE GRAVES, F.L.S. &c. 385

XIX.

The Principles of Physiology applied to the Preservation of Health, and to the Improvement of Physical and Mental Education. By ANDREW COMBE, M.D. . . . . . 388

*Reviews continued.*

XX.

The Gardener's Dictionary, &c. By PHILIP MILLER, F.R.S. &c. . 390

XXI.

Observations on the present System of Medical Education, with a View to Medical Reform. By A LICENTIATE OF THE ROYAL COLLEGE OF PHYSICIANS OF LONDON. . . . . 391

XXII.

The Magazine of Botany and Gardening, British and Foreign. Edited by JAMES RENNIE, M.A. . . . . 394

XXIII.

Vindiciæ Medicæ; or, a Defence of the College of Physicians . ib.

XXIV.

Medica Sacra; or, Short Expositions of the most important Diseases mentioned in the Sacred Writings. By THOMAS SHAPTER, M.D. . 396

XXV.

Illustrations of the Elementary Forms of Disease. By R. CARSWELL, M.D. 397

XXVI.

The Cyclopædia of Practical Surgery. By WILLIAM BLOXAM, Esq., M.R.C.S. Revised by CHARLES MILLARD, Esq., M.R. .S. . . 398

XXVII.

The Anatomy and Operative Surgery of Inguinal and Femoral Hernia. By VALENTINE FLOOD, A.M. M.D., &c. . . . ib.

XXVIII.

Pharmacopœia Homœopathica. Edidit T. F. QUIN, M.D. . 399

XXIX.

The Signs, Disorders, and Management of Pregnancy; the Treatment to be adopted during and after Confinement; and the Management and Disorders of Children. Written expressly for the Use of Females. By DOUGLAS FOX, F.R.C.S. . . . . . 401

### Original Communications.

Cases extracted from the Note-book of HENRY DAVIES, M.D., Physician to the British Lying-in Hospital, &c. . . . 402

On the Sense of Equilibrium, and on its Disturbance. By H. MAYO, F.R.S. 405

Comparison of the Circular and Flap Operations in amputating the Thigh. By HERBERT MAYO, F.R.S. . . . . 411

Observations on Catarrhal and Catarrho-rheumatic Ophthalmia. By FREDERICK TYRRELL, Esq. . . . . 416

Observations on the Treatment of Bronchocele. By JAMES REID, Esq. 425

A Case, in which a Collection of Fluid and Coagulable Matter, to the amount of several Pints, took place between the Membranes of the Brain 446

Case of Strangulated Hernia, with Adhesions to the Sac. By JOHN VALENTINE, M.R.C.S. . . . . 448

MR. MAYO on Sir CHARLES BELL's Discoveries . . . 451

### COLLECTANEA.

### PATHOLOGY AND PRACTICE.

On the Circular and Flap Operations . . . 452

Condylomata cured by Creosote . . . . 453

In what Dose should Digitalis be given ? . . . 454

Rupture of a Varicose Tumour in the Vagina during Labour . 455

Sneezing . . . . . . 456

Case of Disease of the Sympathetic Nerve . . . 457

Treatment of the Venereal Disease in the Hospital of the 72d Regiment, at the Cape of Good Hope . . . . 459

Deep-seated Abscess of the Thigh . . . . ib.

On the Death of New-born Infants depending on Anormal States of the Umbilical Cord. By Dr. KOHLSCHWETTER . . 462

Efficacy of the Extract of Belladonna in Strangulated Hernia . 465

On the Division of the Tendo Achillis as a Means of curing Club-foot 466

Conversion of the Right Lung into an Encephaloid Structure . 468

Chronic Disorders of the Heart . . . . 471

Pathology of the Fœtus . . . . 473

Phlegmasia Dolens in Men . . . . 475

Hypertrophy of the Mammæ . . . . 477

Triple Quotidian Ague . . . . 479

### MISCELLANEOUS.

King James's Counterblaste to Tobacco . . . ib.

State of Drugs in London . . . . 481

Mr. Richmond's Musical Performance . . . 483

Theory of Animal Magnetism . . . . 484

State Medicine in Germany . . . . . 486

John Hunter.—Symptoms of Pain in the Horse . . . 489

Small Hospitals.—Pure Tannin . . . . 490

On the Action of Chlorine on Metallic Iodides . . . 491

### Medical Politics and Intelligence.

What can the Legislature do for us ? . . . . 492

Self-supporting Dispensaries . . . . 494

Meteorological Register. Notices . . . . 496

---

**Advertising Sheet—(see End of the Number.)**

